# Producing human ceramide-NS by metabolic engineering using yeast *Saccharomyces cerevisiae*

**DOI:** 10.1038/srep16319

**Published:** 2015-11-17

**Authors:** Suguru Murakami, Toshi Shimamoto, Hideaki Nagano, Masahiro Tsuruno, Hiroaki Okuhara, Haruyo Hatanaka, Hiromasa Tojo, Yukiko Kodama, Kouichi Funato

**Affiliations:** 1 Department of Biofunctional Science and Technology, Graduate School of Biosphere Science, Hiroshima University, Hiroshima, 739-8528, Japan; 2 Suntory World Research Center, Kyoto 619-0284, Japan; 3 Department of Biophysics and Biochemistry, Osaka University Graduate School of Medicine, Suita 565-0871, Japan

## Abstract

Ceramide is one of the most important intercellular components responsible for the barrier and moisture retention functions of the skin. Because of the risks involved with using products of animal origin and the low productivity of plants, the availability of ceramides is currently limited. In this study, we successfully developed a system that produces sphingosine-containing human ceramide-NS in the yeast *Saccharomyces cerevisiae* by eliminating the genes for yeast sphingolipid hydroxylases (encoded by *SUR2* and *SCS7*) and introducing the gene for a human sphingolipid desaturase (encoded by *DES1*). The inactivation of the ceramidase gene *YDC1*, overexpression of the inositol phosphosphingolipid phospholipase C gene *ISC1*, and endoplasmic reticulum localization of the DES1 gene product resulted in enhanced production of ceramide-NS. The engineered yeast strains can serve as hosts not only for providing a sustainable source of ceramide-NS but also for developing further systems to produce sphingosine-containing sphingolipids.

The outermost layer of skin, known as the stratum corneum, retains moisture and forms a barrier to protect the skin against external stimuli^1^,^2^,^3^,^4^. The stratum corneum consists of keratinocytes, natural moisturizing factors, and intercellular lipids. Ceramides make up approximately half of the intercellular lipids and play a crucial role in these functions. For example, a common characteristic of atopic dermatitis and senile xerosis is a decline in the ability to retain moisture, resulting from decreased ceramide levels due to abnormal lipid metabolism^1^,^3^,^5^,^6^,^7^. Moreover, ceramides have been shown to strengthen the skin’s barrier function and to suppress the production of melanin^8^.

Ceramides can be externally supplied^9^,^10^,^11^; hence, they have garnered considerable attention for use in treatments for dry, sensitive skin as well as in cosmetics and health foods. In fact, a number of ceramide-containing cosmetic, food, and supplement products have already been commercialized, and the market for ceramide materials continues to grow^11^. Ceramides derived from animal sources (e.g. cows) have been conventionally used but, because of concerns regarding infectious diseases such as bovine spongiform encephalopathy, are currently being replaced by plant-derived ceramides from rice, wheat, beans, and potatoes. However, the amount of free ceramides in plants is very low and they are difficult to extract and purify. Moreover, previous studies have suggested that the structures of ceramides are important for the moisturizing and barrier functions of the skin^12^,^13^,^14^. This raises the question about whether plant ceramides, which are structurally different from human ceramides, are functional lipids in humans. Therefore, a new production technique that can overcome these problems is desired.

Yeast has been widely and successfully used as a host for the production of useful substances in the fields of cosmetics, health food, and medicine. Further, the relative amount of ceramides in the yeast *S. cerevisiae* (0.5 ~ 2 mg/g yeast)^15^,^16^ is higher than that in plants currently used to produce ceramides, such as soybean and wheat (0.005 ~ 0.05 mg/g; estimated using reported data of glucosylceramide contents and a ratio of ceramide to glucosylceramide)^17^,^18^,^19^.This suggests that *S. cerevisiae* is a potential host for efficient production of human ceramides.

In this study, we developed a system that produces a human ceramide, ceramide-NS containing sphingosine, in the yeast *S. cerevisiae*. *S. cerevisiae* does not synthesize sphingolipids containing sphingosine, which is desaturated at the C4 position of dihydrosphingosine (DHS)^20^,^21^. Instead, it makes phytosphingosine (PHS)-containing sphingolipids. Therefore, in order to produce ceramide-NS effectively, we metabolically engineered the yeast *S. cerevisiae* and introduced the heterogenous *DES* gene encoding a sphingolipid Δ4-desaturase. In addition, by controlling sphingolipid metabolism and the localization of the *DES* gene product in the host yeast, we successfully increased the production of ceramide-NS. To our knowledge, this is the first example of genetically engineered ceramide-NS-producing yeast *S. cerevisiae*.

## Results

### Expression of hDES1 in sur2Δ scs7Δ mutant S. cerevisiae results in production of sphingosine-containing ceramide-NS

**Figure 1:**
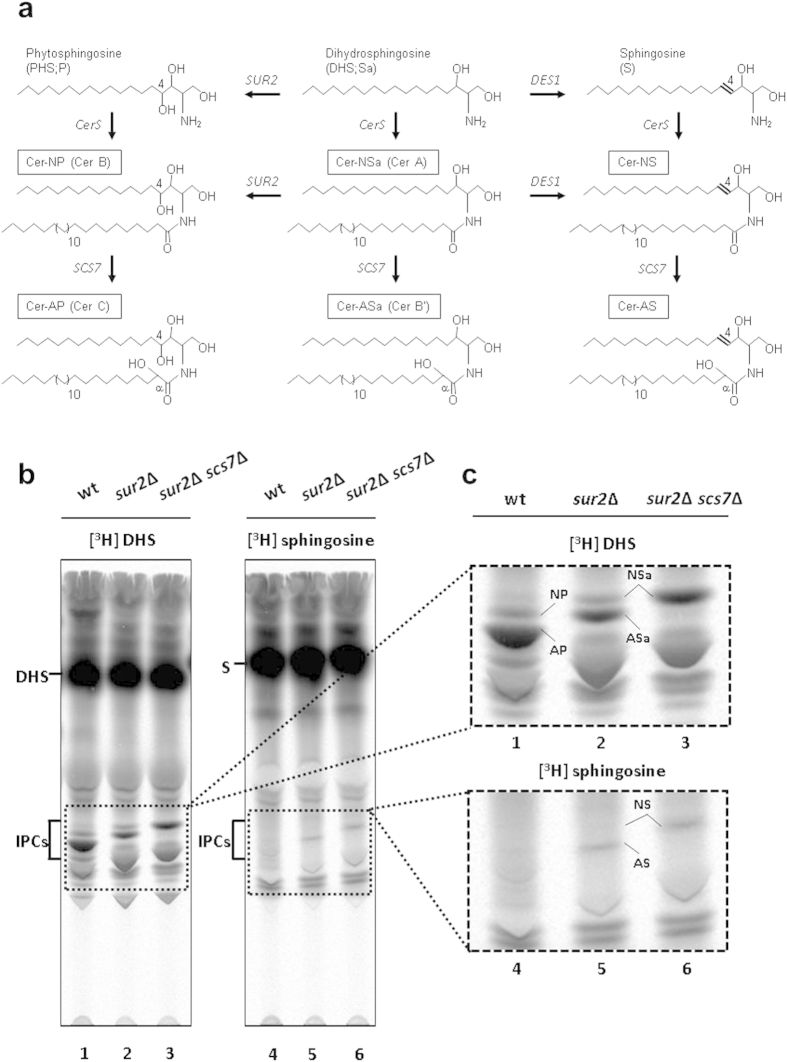
Sphingosine is metabolized to inositolphosphorylceramide in the *Saccharomyces cerevisiae* C4 hydroxylase-deficient sur2Δ mutant. (**a**) Structure of ceramides. The nomenclature of Motta *et al.*^51^ is used; Cer-NSa/Cer-ASa, Cer-NP/Cer-AP, and Cer-NS/Cer-AS contain dihydrosphingosine (DHS, Sa), phytosphingosine (PHS, P), and sphingosine (S), respectively, linked to non-hydroxyl acyl group (N) or alpha-hydroxyl acyl group (A). The denotations in parentheses give the ceramide classification as used in Haak *et al.*^22^. CerS, ceramide synthases; *SUR2*, yeast sphingolipid C4-hydroxylase gene; *SCS7*, yeast fatty acid alpha-hydroxylase gene; *DES1*, sphingolipid Δ4-desaturase gene. Ceramides are converted to inositolphosphorylceramides (IPCs, IPC-NSa, IPC-ASa, IPC-NP, IPC-AP, IPC-NS, IPC-AS) by IPC synthase. (**b**) Cells were labeled with [^3^H]DHS or [^3^H]sphingosine 37 °C for 3h. The labeled lipids were extracted, subjected to mild alkaline hydrolysis and separated by TLC with solvent system I. (**c**) The indicated regions of the TLC image in (**b**) are displayed in an enlarged view.

Since *S. cerevisiae,* the host organism used in this study, does not contain the sphingolipid Δ4-desaturase gene (*DES1*), sphingosine is not synthesized^20^,^21^. Instead, PHS is synthesized through the hydroxylation of C4 of DHS by the yeast sphingolipid C4-hydroxylase gene (*SUR2*) product (Fig. 1a)^22^. DHS and PHS are then amide linked to a very long chain fatty acid (C22–C26) that is almost exclusively C26-fatty acid to yield very long chain dihydroceramides (DHCer, Cer-NSa and Cer-ASa) and phytoceramides (PHCer, Cer-NP and Cer-AP), respectively, by the ceramide synthases Lag1 and Lac1^23^. *S. cerevisiae* further utilizes the *SCS7* gene encoding the sphingolipid fatty acid alpha-hydroxylase Scs7, which synthesizes ceramides containing an alpha-hydroxy fatty acid^22^. The very long chain ceramides formed in the endoplasmic reticulum (ER) are then transported to the Golgi apparatus^24^ and converted to complex sphingolipids such as inositolphosphorylceramide (IPC), mannosyl IPC (MIPC), and mannosyl di-IPC (M(IP)_2_C)^23^. The conversion of ceramide to IPC is catalyzed by the IPC synthase, Aur1, which is encoded by the *AUR1* gene^23^,^25^.

If *S. cerevisiae* has the ability to convert sphingosine to ceramide, it should be able to produce sphingosine-containing human ceramides in a metabolically engineered yeast strain capable of producing sphingosine. Therefore, we first examined whether exogenously delivered [^3^H]sphingosine could be converted into yeast complex sphingolipids. We measured the incorporation of [^3^H]sphingosine or [^3^H]DHS into IPCs in wild-type, *sur2*Δ, and *sur2*Δ *scs7*Δ mutant yeast strains. As shown in Fig. 1b,c (lane 4), only trace amounts of IPC-containing sphingosine was detected in wild-type cells labeled with [^3^H]sphingosine. In the *sur2*Δ background, only sphingolipids, IPC-NSa and IPC-ASa, are synthesized when labeled with [^3^H]DHS (Fig. 1b,c, lane 2). In this background, when the cells were labeled with [^3^H]sphingosine, a significant amount of IPC-AS containing sphingosine and alpha-hydroxyl acyl group was produced (Fig. 1b,c; lane 5), although the conversion of sphingosine to IPC was less efficient than that of DHS (Fig. 1b,c; lanes 4–6 vs. lanes 1–3). In addition, the conversion of sphingosine to IPC-NS containing non-hydroxyl acyl group (Fig. 1b,c; lane 6) was observed in the *sur2*Δ *scs7*Δ mutant strain, in which only IPC-NSa is synthesized (Fig. 1b,c; lane 3). These results indicate that *S. cerevisiae* is capable of synthesizing ceramide-NS/AS from sphingosine.

**Figure 2:**
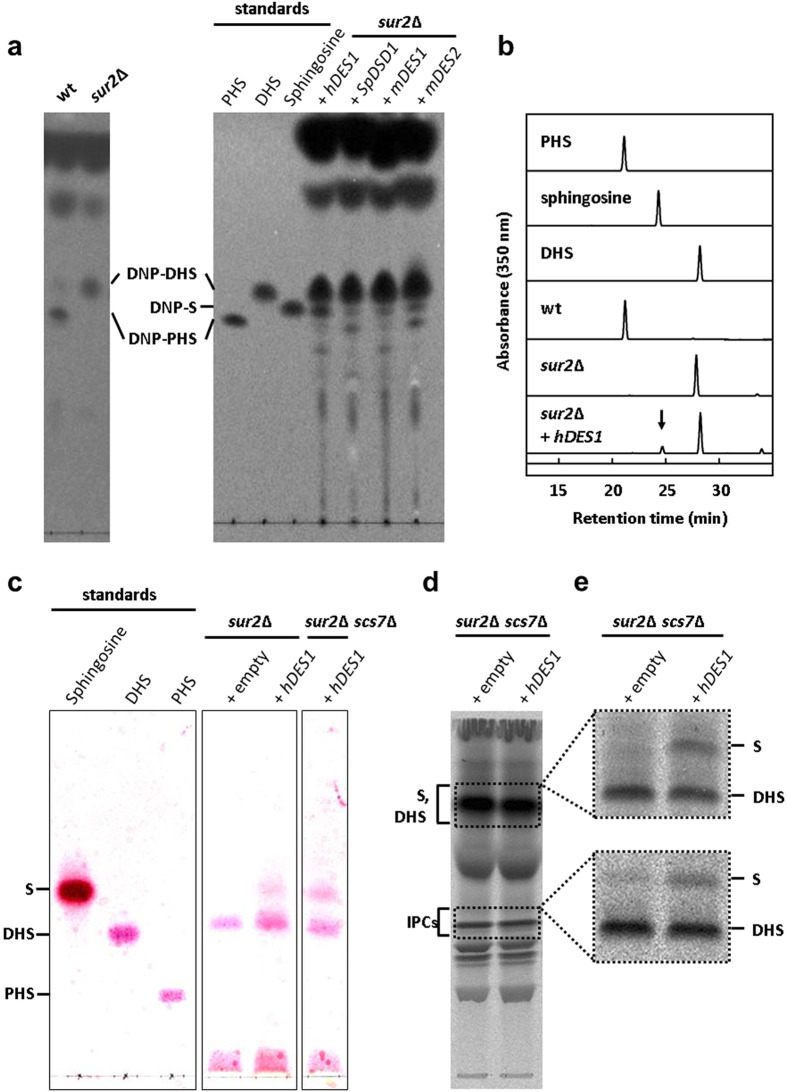
Sphingolipids containing sphingosine are formed in the sur2Δ or sur2Δ scs7Δ yeast mutant strains expressing human DES1. (**a**,**b**) The released sphingoid bases from strong alkaline hydrolysis-treated cells were converted into their DNP derivatives and analyzed by TLC (**a**) and HPLC (**b**) as described in Online Methods. For identification of lipids, the following standards were used: PHS, DHS, and sphingosine (S). (**c**) The lipids were extracted from cells, hydrolyzed by HCl, and separated by TLC with solvent system II. Sphingoid bases were visualized by spraying with 0.5% ninhydrin and heating at 100 °C. (**d**,**e**) Cells were labeled with [^3^H]DHS overnight at 25 °C. The labeled lipids were extracted, subjected to mild alkaline hydrolysis and separated by TLC with solvent system I (**d**). Fractions containing sphingoid bases (DHS, sphingosine) and IPCs in (**d**) were collected from the silica. The fraction containing IPCs but not sphingoid bases was subjected to strong HCl hydrolysis. Samples were separated by TLC with solvent system II (**e**).

Next, to construct the sphingosine-producing strain, we cloned *Homo sapiens DES1* (*hDES1*), *Mus musculus DES1* (*mDES1*) and *DES2* (*mDES2*), and *Schizosaccharomyces pombe DSD1* (*SpDSD1*) into the yeast expression vector pRS426 under the control of the GPD promoter so they could be expressed in the *sur2*Δ mutant strain. It was previously reported that expression of hDES1, mDES1, or mDES2 in *sur2*Δ cells led to the production of sphingolipids containing sphingosine^20^. Consistent with this report, we detected sphingolipid Δ4-desaturase activity in the *sur2*Δ strain expressing these DES proteins (Fig. 2a). The expression of SpDSD1, like mDES2^20^, in the *sur2*Δ strain resulted in the formation of sphingolipids containing PHS in addition to sphingosine, indicating bifunctional desaturase/hydroxylase activity. Because the highest productivity of sphingolipids containing sphingosine was obtained in the *sur2*Δ strain carrying hDES1, which does not produce sphingolipids containing PHS (Fig. 2a), we have chosen hDES1 for further studies.

As shown in Fig. 2b, HPLC analysis of the DNP-derivatized sphingoid bases from the *sur2*Δ strain expressing hDES1 showed a peak corresponding to the authentic DNP-labeled sphingosine. The formation of sphingosine-containing sphingolipids in the *sur2*Δ strain expressing hDES1 was confirmed by the ninhydrin assay (Fig. 2c). A *sur2*Δ *scs7*Δ mutant strain expressing hDES1 also produced sphingosine-containing sphingolipids. Moreover, we detected free sphingosine in the *sur2*Δ *scs7*Δ mutant strain expressing hDES1 when labeled with [^3^H]DHS (Fig. 2d,e, upper). In the same strain, [^3^H]-labeled IPCs yielded sphingosine upon strong HCl hydrolysis (Fig. 2e, lower), showing that the IPCs contain sphingosine and that this yeast strain can produce sphingosine-containing ceramides.

**Figure 3:**
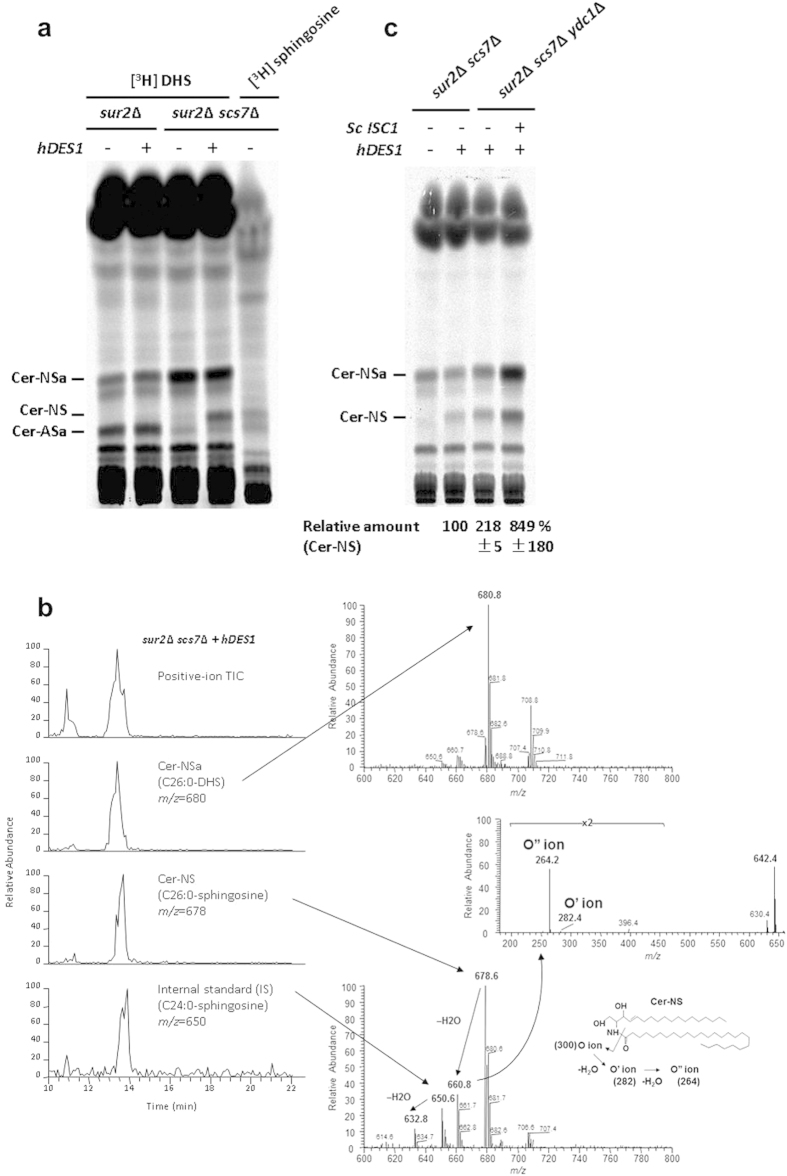
Ceramide-NS is formed in the sur2Δ scs7Δ mutant expressing human DES1. (**a**,**c**) Cells were labeled with [^3^H]DHS overnight at 25 °C. The labeled lipids were extracted, subjected to mild alkaline hydrolysis, spotted on borate-impregnated TLC plates, and developed with solvent system III. Radioactivity of ceramide-NS in (**c**) was quantified and expressed as the relative amount to that in *sur2*Δ *scs7*Δ mutant expressing human DES1.Data represent the average of two independent experiments with error bars denoting the range of the two experiments. (**b**) HPLC/ion-trap mass spectroscopy of ceramides in the *sur2*Δ *scs7*Δ mutant expressing human DES1. (Left) Total ion current (TIC) and ion chromatograms of the most intense signals of Cer-NSa (C26:0), Cer-NS (C26:0), and internal standard Cer-NS (C24:0) in the positive-ion mode. (Right) Mass spectra of Cer-NSa (upper panel) and Cer-NS (lower panel). The lower inset shows the fragmentation patterns of Cer-NS at the amide linkage^12^. A MS-MS spectrum of the m/z 660.8 ions (middle panel).

In order to confirm that the *sur2*Δ *scs7*Δ mutant strain expressing hDES1 can make ceramides containing sphingosine, we labeled the cells with [^3^H]DHS and subsequently analyzed for incorporation of [^3^H]DHS into ceramides. Thin layer chromatography (TLC) analysis showed that Cer-NS, which contains sphingosine and a non-hydroxyl acyl group, was detected in the *sur2*Δ *scs7*Δ mutant strain expressing hDES1, whereas it could not be detected in the *sur2*Δ mutant or strains not expressing hDES1 (Fig. 3a). The ceramide structure was confirmed by mass spectrometry (Fig. 3b). The ceramides observed in the *sur2*Δ *scs7*Δ mutant strain expressing hDES1 were C26:0-CerNSa (*m/z* = 680) and C26:0-CerNS (*m/z* = 678). Mass spectrometry (MS/MS) analysis of ion *m/z* 678 showed the presence of sphingosine ions (*m/z* 264, 282, and 300) arising from cleavage at the 2-amide linkage^12^. These results indicate that expressing hDES1 in the *sur2*Δ *scs7*Δ mutant strain results in production of C26:0-CerNS.

We set out to improve the production of ceramide-NS in *S. cerevisiae* by metabolic engineering. Since *YDC1* encodes an alkaline ceramidase that deacylates DHCer to yield a free fatty acid and DHS^26^, we reasoned that deletion of *YDC1* would inhibit ceramide degradation and increase production of ceramides. Therefore, we created a *sur2*Δ *scs7*Δ *ydc1*Δ triple mutant strain and subsequently introduced *hDES1* gene. We found that when yeast cells were labeled with [^3^H]DHS, the level of ceramide-NS was increased about 2-fold in the *sur2*Δ *scs7*Δ *ydc1*Δ triple mutant compared to the level of ceramide-NS in the *sur2*Δ *scs7*Δ double mutant strain (Fig. 3c). In addition, we chose to overexpress the Isc1 (inositol phosphosphingolipid-phospholipase C1) protein to test its effect on ceramide levels, as Isc1 hydrolyzes complex sphingolipids to produce ceramides^27^. As expected, ceramide levels were drastically increased in the *sur2*Δ *scs7*Δ *ydc1*Δ strain expressing Isc1, and the level of ceramide-NS was about 4-fold higher in Isc1-expressing cells than in cells not expressing Isc1 (Fig. 3c).

### Expression of hDES1 results in enhancement of aureobasidin A sensitivity

**Figure 4:**
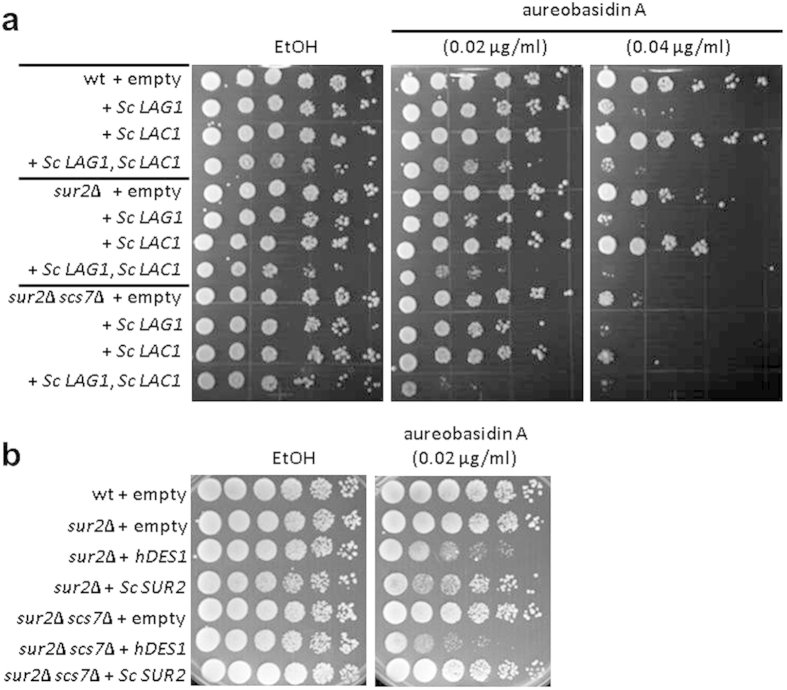
Overexpression of human DES1 enhances aureobasidin A sensitivity of the sur2Δ scs7Δ mutant strain. (**a**,**b**) Fivefold serial dilutions of cells were spotted onto SD plates supplemented with ethanol as a vehicle, 0.02 μg/ml AbA (**a**,**b**) or 0.04 μg/ml AbA (**a**).

Inhibition of Aur1 by aureobasidin A (AbA) or repression of *AUR1* expression leads to cell death or growth inhibition^28^,^29^,^30^,^31^. It has been suggested this is due to the accumulation of ceramides and a reduction in complex sphingolipids. In a previous study using strains of the BY4741 background, it was reported that the deletion of *SUR2* causes a reduction in complex sphingolipid levels, while attenuating growth inhibition under an *AUR1*-repressive condition^28^. Moreover, we observed reduced levels of complex sphingolipids when *SUR2* was deleted in RH448 or RH6082 cells of a different background^32^,^33^. However, *SUR2* deletion aggravated the growth inhibition caused by AbA (Fig. 4a). Although the reason for the discrepancy between studies is unclear, it could be related to different strain backgrounds, cultivation (YPD; rich medium containing yeast extract, peptone, dextrose, SD; synthetic defined minimal medium), or experimental systems. In addition, we found that the *sur2*Δ *scs7*Δ double mutant strain shows more severe growth inhibition than the *sur2*Δ strain (Fig. 4a). This is consistent with previous results with the repression of *AUR1* expression^28^. Given that the AbA-induced growth inhibition is due to ceramide accumulation, these results suggest that DHCer containing non-hydroxyl acyl groups are more toxic than PHCer, since the double deletion of *SUR2* and *SCS7* causes accumulation of Cer-NSa (Fig. 3a). In order to support this hypothesis, we overexpressed ceramide synthases (Lag1 and Lac1) and examined its effects on AbA-induced growth inhibition. In all strains tested (wild-type, *sur2*Δ, *sur2*Δ *scs7*Δ), the overexpression of ceramide synthases aggravated the growth inhibition by AbA. The most severe growth inhibition was observed in the *sur2*Δ *scs7*Δ strain expressing both Lag1 and Lac1. These results suggest that AbA-induced growth inhibition depends on the ability to synthesize ceramides.

We next wanted to know whether replacing DHCer by ceramide would affect the growth inhibition caused by AbA. As shown in Fig. 4b, the expression of hDES1 in the *sur2*Δ or *sur2*Δ *scs7*Δ strains caused more potent growth inhibition than caused by no expression of hDES1 or expression of Sur2. These results suggest that ceramides containing sphingosine are more toxic than DHCer or PHCer, and the growth inhibition assay could be used as a predictive analytical tool for the further development of systems to produce ceramide-NS efficiently.

### ER-retention sequence KKEK fused to the C-terminus of hDES1 enhances production of ceramide-NS

**Figure 5:**
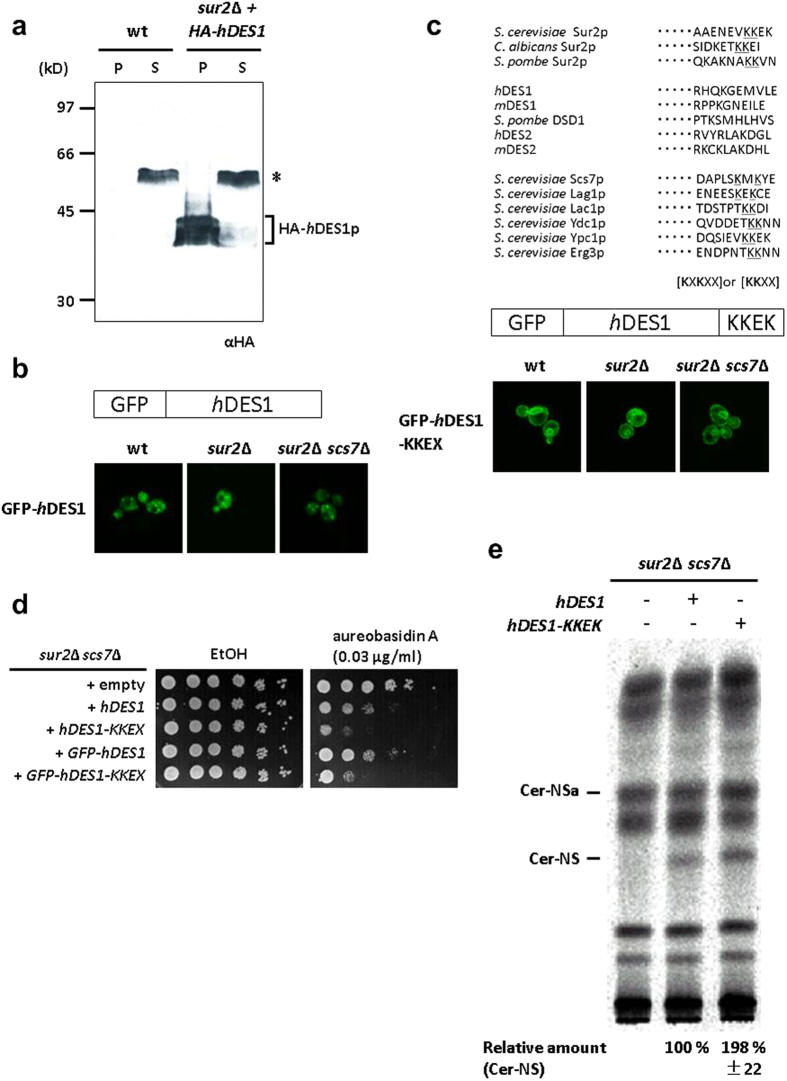
Human DES1 carrying a C-terminal di-lysine motif enhances production of ceramide-NS in the sur2Δ scs7Δ mutant strain. (**a**) Cell lysates were prepared from wild-type or *sur2*Δ mutant strains expressing HA-tagged human DES1, and centrifuged at 100,000 × *g* for 60 min. The pellet (P) and soluble (S) fractions were subjected to SDS-PAGE followed by Western blotting. An asterisk indicates a nonspecific band. (**b**,**c**) Wild-type, *sur2*Δ, and *sur2*Δ *scs7*Δ mutant strains expressing GFP-human DES1 (**b**) or GFP-human DES1 carrying a C-terminal di-lysine motif (**c**) were observed by fluorescence microscopy. C-terminal amino acid sequences from yeast Sur2p, various DES1 and *S. cerevisiae* enzymes involved in lipid metabolism. Di-lysine ER retrieval signals, KKXX or KXKXX^39^, are underlined (**c**). (**d**) Five-fold serial dilutions of cells were spotted onto SD plates supplemented with ethanol as a vehicle and 0.03 μg/ml AbA. (**e**) Cells were labeled with [^3^H]DHS overnight at 25 °C. The labeled lipids were extracted and separated as described in Fig. 3a. Radioactivity of ceramide-NS was quantified and expressed as the relative amount to that in *sur2*Δ *scs7*Δ mutant expressing human DES1. Data represent the average of two independent experiments with error bars denoting the range of the two experiments.

As hDES1 has multiple putative transmembrane segments^34^, it is assumed to be an integral membrane protein. In fact, sphingolipid Δ4-desaturase activity and hDES1 were detected in membrane fractions from human neuroblastoma^35^ and HeLa cells^36^, respectively. Moreover, we observed that the majority of hemagglutinin (HA)-tagged hDES1 was found in the membrane pellet fraction when expressed in the *sur2*Δ strain (Fig. 5a). As sphingolipid Δ4-desaturase proteins from other species, including monkey DES1^36^ and SpDSD1^37^, have been shown to be localized to the ER and the sphingolipid Δ4-desaturases substrates (DHS and DHCer) are likewise synthesized in the ER, it is conceivable that hDES1 also localizes to the ER membrane. However, when GFP-tagged hDES1 (GFP-hDES1) was expressed in yeast cells, the fluorescence exhibited punctate Golgi structures, which are different from the ring-like structures of the ER^38^. This Golgi localization of GFP-hDES1 might be due to the lack of a di-lysine motif in its C-terminus. Di-lysine motifs, such as KKXX and KXKXX, are commonly found in ER membrane proteins involved in yeast lipid metabolism (Fig. 5c, upper) and contribute to their localization through the interaction with the COPI coat that mediates retrograde trafficking from the Golgi to the ER^39^,^40^,^41^. Therefore, we introduced the ER retention C-terminal di-lysine motif of *SUR2* (KKEK) at the C-terminus of GFP-hDES1 and observed its localization by fluorescence microscopy. As shown in Fig. 5c, the GFP-hDES1 with the KKEK amino acid sequence (GFP-hDES1-KKEK) was found in ring-like ER structures. The access of a substrate to an enzyme could potentially be increased when they are localized to the same site so the ER localization of GFP-hDES1-KKEK raises the possibility that introduction of the KKEK signal leads to an increase in production of ceramides containing sphingosine. Thus, we examined the effect of the KKEK motif on hDES1 on AbA sensitivity. The *sur2*Δ *scs7*Δ strains expressing GFP-hDES1-KKEK or hDES1-KKEK were more sensitive to AbA compared to the strains expressing GFP-hDES1 and hDES1 (Fig. 5d). We next tested whether the introduction of the KKEK motif affects production of ceramide-NS in the *sur2*Δ *scs7*Δ strain. As shown in Fig. 5e, cells expressing hDES1-KKEK produced about 2-fold more ceramide-NS than cells expressing hDES1. These results indicate that ER localization of hDES1 causes increased ceramide-NS production.

## Discussion

In this study, *S. cerevisiae* was engineered to produce human sphingosine-containing ceramide. Deletions of the *SUR2* and *SCS7* genes and the introduction of *hDES1* into *S. cerevisiae* resulted in the production of ceramide-NS, which contains sphingosine and a non-hydroxyl acyl group. Moreover, the deletion of *YDC1* and overexpression of *ISC1* were found to be efficient in elevating the production of ceramide-NS. Furthermore, the localization of hDES1 to the ER by the introduction of a di-lysine ER retention signal led to the increased production of ceramide-NS.

Although the *sur2*Δ *scs7*Δ double mutant strain producing sphingosine-containing sphingolipids exhibits normal cell morphology and size and the same growth rate as the wild-type cells (data not shown), the engineered strain was hypersensitive to AbA. Because AbA sensitivity was positively correlated with the ability to synthesize ceramides, it is likely that the increased AbA sensitivity is due to the accumulation of sphingosine-containing ceramides. The mechanism for ceramide-triggered growth inhibition or cell death remains unclear, but ceramides containing sphingosine appear to have more potent cytotoxic activity than ceramides containing either DHS or PHS. Interestingly, the cells carrying *LAG1* showed higher sensitivity to AbA than those carrying *LAC1*. This may be due to the specific role of Lag1 in ceramide synthesis or in lifespan of yeast^42^. How ceramides regulate lifespan remains unknown, but a recent study showed that reducing the rate of sphingolipid synthesis increases yeast lifespan by Sch9-dependent and –independent mechanisms^43^. The high toxicity of sphingosine-containing ceramide also suggests that there may be a limitation for production of ceramide-NS in yeast. However, understanding the mechanism of ceramide-induced cell toxicity may help to improve the limitations and efficiency of ceramide-NS production.

Like PHCer, ceramides containing sphingosine are converted into a complex sphingolipid, IPC, through a sphingolipid biosynthetic pathway of yeast, causing a decrease in ceramide levels. This unwanted flux to complex sphingolipids is mediated by the action of IPC synthase, Aur1^25^. Aur1 is an essential protein and the deletion of the *AUR1* gene is lethal for the yeast. The lethality of *aur1∆* is thought to be because of the accumulation of ceramides and the reduction in complex sphingolipid levels^28^,^29^,^30^,^31^. We have previously shown that reduced complex sphingolipids induce metacaspase-dependent apoptotic cell death^30^. However, an *lcb1∆* mutant carrying the *SLC1-1* suppressor gene, which is viable even in the absence of all sphingolipids, was isolated^44^. It was also shown that in a strain containing only short-chain ceramides, the deletion of *AUR1* is not lethal^29^. These results suggest that Aur1 is not essential for yeast cell viability under certain conditions. Thus, by eliminating or modifying apoptotic signals for death induced by the loss of Aur1 function, it might be possible to create a new strain that lacks all complex sphingolipids but is still capable of synthesizing long-chain ceramides. The development of such a strain will lead to improved ceramide-NS yields.

In conclusion, we have constructed a yeast *S. cerevisiae* strain capable of synthesizing ceramide-NS. Since ceramides play a critical role in maintaining the permeability barrier function of the skin, the yeast-derived human ceramide-NS could be used for clinical applications to improve the impaired barrier function seen in several skin diseases including atopic dermatitis. In addition, the yeast strain that we engineered can be used not only as a source of ceramide-NS but also as a potential host to develop systems that produce human complex sphingolipids such as sphingomyelin and gangliosides in yeast.

## Methods

### Plasmids, Strains and Yeast Cultivations

Plasmids are listed in Supplementary Table S1. All yeast strains are derivatives of RH6082 (*Mat a ura3*, *his3*, *leu2*, *lys2*, *trp1*, *bar1-1*) and listed in Supplementary Table S2. DNA manipulation and yeast construction were carried out by standard techniques described elsewhere^45^,^46^. Wild type, deletion mutants, and strains carrying plasmids were maintained in SD minimal medium supplemented with the appropriate nutrients. To test the sensitivity of strains to AbA, 5-fold serial dilutions of cells were made in sterile water and spotted onto SD plates containing the indicated concentrations of AbA^30^.

### *In Vivo* Labeling with [^3^H]DHS and [^3^H]sphingosine

Labeling of sphingolipids with [^3^H]DHS or [^3^H]sphingosine was performed as described previously^30^,^45^. In brief, cells grown overnight were resuspended in SD medium and labeled with 4–10 μCi of [^3^H]DHS or [^3^H]sphingosine for the indicated times. The reaction was stopped by adding 10 mM NaF and 10 mM NaN_3_; the cells were washed with cold water, and lipids were extracted with chloroform-methanol-water (10:10:3, v/v/v). The samples were subjected to mild alkaline hydrolysis (0.2 M NaOH, 90 min) to deacylate glycerophospholipids, desalted by partitioning with n-butanol, and dried under nitrogen. The lipids were analyzed by TLC using solvent system I, chloroform-methanol-4.2 N ammonium hydroxide (9:7:2, v/v/v) for sphingolipids^30^,^45^ and visualized on an FLA-7000 system (Fujifilm, Japan). The fractions containing sphingoid bases or inositolphosphorylceramide (IPC) were collected from the TLC plates by scraping and eluting with chloroform-methanol (1:1, v/v). The lipid extract was dried, subjected to strong HCl hydrolysis^24^, and analyzed by TLC using solvent system II, chloroform-methanol-2.5 N ammonium hydroxide (40:10:1, v/v/v) for sphingoid bases as described^22^.

For ceramide analysis, radioactively labeled lipids were spotted on borate-impregnated TLC plates, and developed with solvent system III, chloroform-methanol (9:1, v/v)^47^. After the development, the lipids were visualized and quantified on an FLA-7000 system (Fujifilm, Japan).

### Sphingoid Base Analysis

Sphingoid base analysis by HPLC was carried out by a modified method of Sperling *et al.*^48^. Cells were directly subjected to strong alkaline hydrolysis and the released sphingoid bases were extracted by separation into layers with chloroform-1, 4-dioxane-water (8:3:8, v/v/v). The organic layers were washed with equal amounts of 0.1 M KOH and 0.5 M KCl. The sphingoid bases were then converted to DNP derivatives, dried, spotted on TLC plates, and developed with solvent system III. Derivatized sphingoid bases were observed as yellow spots (dark blue under UV radiation), eluted from the silica gel with chloroform-methanol (2:1, v/v), and then separated into layers with chloroform-methanol-0.1 M KOH (2:1:1, v/v/v). The organic layers were dried and DNP-derivatized sphingoid bases were analyzed by HPLC. HPLC was performed on a silica gel ODS column, eluting with a linear gradient of 80% methanol-acetonitrile-2-propanol (10:3:1, v/v/v) and 20% water to 0% water (flow rate 1 ml/min, 40 min), and UV absorption at 350 nm was monitored.

For detection of sphingoid base by ninhydrin, lipids were extracted from cells with chloroform-methanol-water (10:10:3, v/v/v), subjected to strong HCl hydrolysis^24^, spotted on TLC plates, and developed with solvent system II. The TLC plates were sprayed with 0.5% ninhydrin in 1-butanol and incubated at 100 °C for 5–10 min to visualize the sphingoid bases^22^.

### HPLC/Mass Spectrometric Analysis

Molecular species of ceramide were identified by normal-phase HPLC/ion-trap mass spectrometry^12^,^49^. Lipid extracts were prepared from double *sur2*Δ *scs7*Δ mutant cells transformed with pRS426-*hDES1* plasmid that contains the *hDES1* gene and mixed with appropriate amounts of N-lignoceroyl-(C24:0) sphingosine as an internal standard. The lipids were dried and dissolved in hexane/2-propanol (3:2). After mild alkaline hydrolysis, the samples were subjected to HPLC/mass spectrometry. Separation and identification of ceramides were performed as described^12^.

### Western Blot Analysis

Protein extraction, membrane (pellet, P) and cytosolic (supernatant, S) fraction preparation, SDS-PAGE, and Western blotting were performed as described previously^50^.

### Fluorescence Microscopy

To assess the localization of the hDES1 protein, pRS426-*GFP-hDES1* or pRS426-*GFP-hDES1-KKEK* was transformed into yeast cells. The transformed cells were grown in SD medium lacking uracil and viewed under a fluorescence microscope.

## Additional Information

**How to cite this article**: Murakami, S. *et al.* Producing human ceramide-NS by metabolic engineering using yeast *Saccharomyces cerevisiae*. *Sci. Rep.* **5**, 16319; doi: 10.1038/srep16319 (2015).

## Supplementary Material

Supplementary Information
