# Supplementary material for: Producing human ceramide-NS by metabolic engineering using yeast Saccharomyces cerevisiae
Source: Sci Rep. 2015 Nov 17;5:16319. doi: 10.1038/srep16319 (PMC4647206; doi:10.1038/srep16319)
Supplement: Supplementary Information [file srep16319-s1.pdf]

Supplementary information for

“Producing human ceramide-NS by metabolic engineering using yeast *Saccharomyces cerevisiae*”

Table S1. Plasmids used in this study.

Name	Relevant information	Source
pRS426	2μ, GPD promoter, *URA3*	Mumberg et al., Gene (1995) 156, 119-22
pRS426-*hDES1*	2μ, GPD promoter, *URA3*	This study
pRS426-*2HA-hDES1*	2μ, GPD promoter, *URA3*	This study
pRS426-*GFP-hDES1*	2μ, GPD promoter, *URA3*	This study
pRS426-*hDES1-KKEK*	2μ, GPD promoter, *URA3*	This study
pRS426-*GFP-hDES1-KKEK*	2μ, GPD promoter, *URA3*	This study
pRS426-*SpDSD1*	2μ, GPD promoter, *URA3*	This study
pRS426-*mDES1*	2μ, GPD promoter, *URA3*	This study
pRS426-*mDES2*	2μ, GPD promoter, *URA3*	This study
pRS426-*ScSUR2*	2μ, GPD promoter, *URA3*	This study
pRS424	2μ, GPD promoter, *TRP1*	Mumberg et al., Gene (1995) 156, 119-22
pRS424-*ScLAC1*	2μ, GPD promoter, *TRP1*	This study
pRS423	2μ, GPD promoter, *HIS3*	Mumberg et al., Gene (1995) 156, 119-22
pRS423-*ScLAG1*	2μ, GPD promoter, *HIS3*	This study
pYE22m	2μ, *TDH3* promoter, *TRP1*	Sawai-Hatanaka et al., Biosci. Biotechnol. Biochem. (1995) 59, 1221-8
pYE22m-*ScISC1*	2μ, *TDH3* promoter, *TRP1*	This study

Table S2. Strains used in this study.

Name	Genotype	Source or Reference	Figure
RH6082 (FKY113)	*MAT*a *ura3, his3, leu2, lys2, trp1, bar1-1*	Kajiwara et al., Mol Biol Cell (2008) 19, 2069-82	1B-C, 2A-B, 4A-B, 5A-C
FKY268	*MAT*a *sur2Δ::LEU2, ura3, his3, leu2, lys2, trp1, bar1-1*	Ikeda et al., J. Cell Sci. (2015) 1282, 2454-67	1B-C, 2A-C, 3A, 4A-B, 5A-C
FKY277	*MAT*a *scs7Δ::ura3 sur2Δ::LEU2 ura3 his3 leu2 lys2 trp1 bar1-1*	This study	1B-C, 2C-E, 3A-B
FKY300	*MAT*a *scs7Δ::KanMX sur2Δ::LEU2 ura3 his3 leu2 lys2 trp1 bar1-1*	This study	3C, 4A-B, 5B-E
FKY301	*MAT*a *scs7Δ::KanMX sur2Δ::LEU2 ydc1Δ::HIS3 ura3 his3 leu2 lys2 trp1 bar1-1*	This study	3C
